# Barriers to Preparing and Cooking Vegetables Are Associated with Decreased Home Availability of Vegetables in Low-Income Households

**DOI:** 10.3390/nu12061823

**Published:** 2020-06-18

**Authors:** Matthew J. Landry, Marissa Burgermaster, Alexandra E. van den Berg, Fiona M. Asigbee, Sarvenaz Vandyousefi, Reem Ghaddar, Matthew R. Jeans, Adelyn Yau, Jaimie N. Davis

**Affiliations:** 1 Department of Nutritional Sciences, College of Natural Sciences, University of Texas at Austin, Austin, TX 78712, USA; marissa.burgermaster@austin.utexas.edu (M.B.); fiona.asigbee@utexas.edu (F.M.A.); Sarvenaz.Vandyousefi@nyulangone.org (S.V.); reemghaddar94@gmail.com (R.G.); mjeans@utexas.edu (M.R.J.); adelynyau@utexas.edu (A.Y.); jaimie.davis@austin.utexas.edu (J.N.D.); 2 Department of Population Health, Dell Medical School, University of Texas at Austin, Austin, TX 78712, USA; 3 Division of Health Promotion and Behavioral Sciences, Michael and Susan Dell Center for Healthy Living, School of Public Health in Austin, University of Texas Health Science Center at Houston (UT-Health), Austin, TX 78701, USA; Alexandra.E.VanDenBerg@uth.tmc.edu

**Keywords:** food security, food insecurity, vegetable availability, barriers, low-income

## Abstract

Knowing which barriers to buying and preparing/cooking vegetables at home are linked with the home availability of vegetables and how food-security status impacts this relationship will facilitate the tailoring of future public health interventions. Baseline data were used from an elementary-school-based intervention. Data on household food-security status, availability of vegetables at home, and barriers to buying and preparing/cooking vegetables were collected from 1942 parents. Differences between food-secure and food-insecure households were examined for barriers to buying and preparing/cooking vegetables. Mixed-effects linear regression was used to estimate the associations between barriers to buying and preparing/cooking vegetables and food-security status on the home availability of vegetables. Food insecurity was reported in 27% of households. Food-insecure households were significantly more likely to report barriers to buying and preparing/cooking vegetables. The barriers to purchasing/cooking vegetables score was associated with a decrease in the home availability of vegetables score (β = −0.77; 95% CI: −0.88, −0.65; *p* < 0.001). Compared to food-secure households, food-insecure households were 15% less likely to have home vegetable availability (β = −1.18; 95% CI: −1.45, −0.92; *p* < 0.001). Although home availability of vegetables does not guarantee consumption, this study identified specific barriers that were associated with availability that can be targeted in future interventions seeking to improve vegetable consumption in the homes of low-income families.

## 1. Introduction

The 2015–2020 *Dietary Guidelines for Americans* recommends that Americans consume more vegetables as part of an overall healthy dietary pattern [1]. A diet rich in vegetables can protect against diet-related chronic diseases, including heart disease, type 2 diabetes, obesity, and some cancers [1]. Despite the established health benefits, only 13% of Americans aged one and older meet the vegetable recommendations [1]. In particular, vegetable consumption in children and young adolescents falls well below recommended intakes [1].

Parents and other caregivers exert considerable control over the foods that younger children eat; even as adolescents gain greater autonomy over their dietary choices, the home environment plays a significant role in dietary intake [2,3,4]. Numerous demographic, psychosocial, behavioral, and socio-environmental factors have been identified as correlates of children’s vegetable consumption [5,6,7,8,9,10]. Higher vegetable consumption during childhood is associated with healthier eating behaviors over a lifetime [11]; therefore, research identifying ways to promote vegetable consumption during childhood is warranted.

There is a direct correlation between food insecurity in households and decreased intake of vegetables among children [7,12,13]. The US Department of Agriculture (USDA) describes food security as “access by all people at all times to enough food for an active, healthy life” [14]. A lack of the assured ability to acquire acceptable foods in socially acceptable ways is deemed as food insecurity. Food security involves the intersection of four food-system domains or dimensions: availability, access, utilization, and stability [15,16]. Food-insecure households may experience increased barriers to one of these domains, which, in turn, can impact purchasing decisions and dietary consumption.

Availability refers to the ability to obtain quality foods to be consumed [17]. Access is multidimensional and includes physical access to stores or other purchasing locations (farmers’ markets or mobile markets), affordability and quality of available produce, and access to vegetables that are seasonally and culturally appropriate [18]. Research has shown that when healthier foods, such as fruit and vegetables, are available and accessible for purchase by a household and are available and accessible in the home, children have higher intakes of vegetables [12,19,20,21]. The utilization domain of food security has traditionally been overlooked as research has focused on community- or policy-level barriers to availability and access [22]. Utilization incorporates all household practices and individual behaviors in the transformation of food into meals, including planning, management, selection of foods, preparation, and cooking skills [15,16]. In some food-insecure households, barriers to utilization, such as limited preparation/cooking knowledge and skills or perceived time constraints, can result in decreased consumption even when vegetables are physically and economically accessible [23].

As children’s consumption of vegetables is associated with the home availability of vegetables, a greater understanding of the barriers leading to decreased home availability is needed. Knowledge of how barriers both outside and inside the home environment perpetuate food insecurity would facilitate the tailoring of future public health interventions. This study aimed to examine the association of barriers to buying and preparing/cooking vegetables with the home availability of vegetables and how this relationship differs according to food-security status.

## 2. Materials and Methods

### 2.1. Description of Study

Cross-sectional, baseline parent data from TX Sprouts, a cluster-randomized school-based gardening, cooking, and nutrition intervention, were used. TX Sprouts targeted 3rd to 5th grade students and their parents from 16 elementary schools in the Greater Austin area. Methods for the clinical trial are published elsewhere [24]. Briefly, schools were randomized into one of two groups, namely TX Sprouts intervention (*n* = 8 schools) and delayed intervention (*n* = 8 schools), and data collection occurred in three waves between 2016 and 2019. Schools included in the trial had to meet the following inclusion criteria: (1) a high proportion of Hispanic children (>50%), (2) a high proportion of children participating in the free and reduced lunch (FRL) program (>50%), (3) located within 60 miles of the University of Texas at Austin campus, and (4) no existing garden or gardening program. The trial is registered at ClinicalTrials.gov (NCT02668744).

### 2.2. Study Recruitment

All 3rd to 5th grade students and parents at the recruited schools were contacted to participate via information tables at “Back to School” and “Meet the Teacher” evening events, flyers sent home with students, and teachers making class announcements.

### 2.3. Institutional Review Board

Written informed consent was obtained from all parents, and assent was obtained from each student. Both consent and assent were required for inclusion in the study. This study was conducted according to the guidelines laid down in the Declaration of Helsinki, and all procedures involving human subjects were approved by the Institutional Review Board of the University of Texas at Austin (IRB#2014-11-0045) and the individual school district review boards.

### 2.4. Data Collection

At baseline, parents completed a self-administered questionnaire packet that included demographic questions and food-security scales. Questionnaires were completed either at “Back to School” or “Meet the Teacher” evening events or sent home with students, completed by a parent, and returned to school with the student. Questionnaires were provided in both English and Spanish. The questionnaires included questions about child demographics, participation in federal food assistance programs, food and meal choice behaviors, barriers to healthy eating habits, and household food security. Parents received a $15 USD gift card to a local grocery store as an incentive for the time spent completing the questionnaire.

### 2.5. Assessment of Food-Security Status

Data on food-security status were collected using the USDA’s 18-item Household Food Security Survey Module [25]. Following USDA procedure to calculate a participant’s food-security status, the number of affirmative answers were counted. An affirmative answer included answering “yes”, “often”, “sometimes”, “almost every month”, or “some months but not every month”. The total number of affirmatives was a respondent’s raw score. Food-security status was categorized as food-secure (raw score: 0–2) or food-insecure (raw score: 3–18) and analyzed as a dichotomous variable.

### 2.6. Availability and Barriers to Buying and Preparing/Cooking Vegetables

Items assessing home vegetable availability, barriers to buying vegetables, and barriers to preparing/cooking vegetables were adapted from items used in a similar school-based gardening intervention by Evans and colleagues [26]. The availability of four vegetable types was assessed (fresh; canned, dried, and frozen; salad; and cut-up vegetables within easy reach). Three items assessed barriers to buying vegetables (example: “The stores near me do not sell fresh vegetables”) and six items assessed barriers to preparing/cooking vegetables (example: “I don’t know how to prepare vegetables”). Full questionnaire items and response options are provided in Table 1.

A continuous variable for the availability of vegetables within the household was created by coding responses (never = 0, some of the time = 1, most of the time = 2, and all of the time = 3) to the four vegetable-type items and summing to 0–12, with higher scores indicating greater availability. Similarly, continuous variables for the barriers to buying vegetables and barriers to preparing/cooking vegetables were created. Affirmative responses to items were coded as 1 and non-affirmative responses were coded as 0. After summing responses for each type of barrier, scores ranged from 0–3 (buying barriers) and 0–6 (preparing/cooking barriers), with higher scores indicative of experiencing greater barriers.

### 2.7. Study Sample

Of the 4239 eligible children at the 16 schools, 3302 children (or 78%) consented to be in the study. Of those who consented, 3135 children (95% of those who consented) completed baseline clinical measures and child surveys and were in the clinical trial. Approximately 92% (or *n* = 2876) of parents completed baseline surveys. Of parents who completed baseline surveys, 68% (*n* = 1942) provided complete data for food security, availability of vegetables, barriers to buying, barriers to preparing/cooking, and their demographics. Missing data were attributable to parents submitting incomplete questionnaires or accidently skipping questions.

### 2.8. Statistical Analysis

Descriptive statistics were used to describe the sample. Chi-squared (χ^2^) tests and univariate linear regression models were used to determine if significant differences existed between the demographic variables of food-secure and food-insecure households. Chi-squared tests were then used to determine whether differences existed between food-secure and food-insecure households in terms of reported barriers to buying and preparing/cooking vegetables. A mixed-effects linear regression was used to examine the associations between the scores for barriers to buying, barriers to preparing/cooking, and food security on the home availability of vegetables score, with random effects at the school level to account for clustering by schools. Interactions between food insecurity and buying and preparing/cooking scores on the home availability of vegetables score were tested. Race/ethnicity, parent education, number of children in the household, and receipt of Supplemental Nutrition Assistance Program (SNAP) benefits were used as covariates. All analyses were conducted using SPSS Statistics for Macintosh, version 24.0 (IBM Corp, Armonk, NY, USA), and an alpha level of *p* = 0.05 was used for significance.

## 3. Results

The analytic parent sample (*n* = 1942) was predominantly female (87%) and primarily Hispanic (63%). Other races/ethnicities that made up the sample were non-Hispanic white (25%), non-Hispanic black (9%), and other races/ethnicities (3%). A child’s mother or father was the primary questionnaire respondent (98%); other respondents were grandparents (2%) or other guardians (<0.5%). On average, households had 2.7 ± 1.1 children. Twenty-seven percent of the sample reported household food insecurity. Thirty-two percent of the sample reported receiving SNAP benefits. Comparing demographic characteristics between food-secure and food-insecure households, race/ethnicity was significantly different (*p* < 0.002). Food-secure households were more likely to be non-Hispanic white or of another ethnicity, whereas food-insecure households were more likely to be of Hispanic or non-Hispanic African American race/ethnicity. Education level was also significantly different between food-secure and food-insecure households (*p* < 0.001). Food-secure parents were more likely to have a high school diploma, some college, or a college degree compared to food-insecure parents. There were no other significant differences in demographic characteristics between food-secure and food-insecure households.

The distributions of two of the three barriers associated with buying vegetables were significantly different between food-secure and food-insecure households (Table 2). Food-insecure households were more likely to report that vegetables are too expensive (*p* < 0.001) and being unable to find quality fruit and vegetables (*p* < 0.001). All distributions for barriers associated with preparing/cooking vegetables were significantly different between food-secure and food-insecure households (Table 2), with food-insecure households more likely to report difficulty using fresh vegetables before they spoil (*p* < 0.001), their family not liking vegetables (*p* = 0.02), not having time to prepare vegetables (*p* < 0.001), not knowing how to prepare vegetables (*p* < 0.001), not having simple quick recipes (*p* < 0.001), and their family not helping with cooking (*p* < 0.001).

Results from a mixed-effects linear regression model of the impact of barriers to buying vegetables and barriers to preparing/cooking vegetables on the home availability of vegetables score within food-secure and food-insecure households are provided in Table 3. Each additional barrier to preparing/cooking vegetables was associated with a −0.77 unit decrease in vegetable availability at home (95% CI: −0.88, −0.65; *p* < 0.001). The barriers to buying vegetables score was not significantly associated with vegetable availability at home. A significant association between food-security status and the availability of home vegetables score was observed (β = −1.18; 95% CI: −1.45, −0.92; *p* < 0.001). Compared to food-secure households, food-insecure households were associated with a 15% lower availability of home vegetables (mean ± SE: 8.32 ± 1.9 versus 7.14 ± 1.9). There was a significant association between race/ethnicity and the home availability of vegetables (*p* < 0.001). Compared to non-Hispanic white households, Hispanic households had 7% lower home availability of vegetables scores (β = −0.56; 95% CI: −1.00, −0.12; *p* = 0.01). Compared to non-Hispanic white households, non-Hispanic African American households had 4% lower home availability of vegetables scores (β = −0.35; 95% CI: −0.64, −0.06; *p* = 0.02) and other race/ethnicity households had 14% lower availability (β = −1.14; 95% CI: −1.76, −0.51; *p* < 0.001).

## 4. Discussion

In this large, cross-sectional examination of home vegetable availability and barriers to buying and preparing vegetables among low-income families in the Greater Austin area of Texas, we found that food-insecure families reported lower vegetable availability and that food-insecure families were more likely to report barriers to buying and preparing/cooking vegetables. However, while barriers to preparing/cooking vegetables were significantly associated with lower vegetable availability, barriers to buying vegetables were not. Although home availability of vegetables does not guarantee consumption, the barriers identified in this study should be considered when designing future interventions for low-income populations.

The home plays a central role in influencing child dietary consumption and may serve as a modifiable target for interventions [27]. There has been evidence that the home availability of vegetables results in a greater intake of vegetables among children [20]. Although the availability of vegetables does not guarantee consumption, children within food-insecure households may have a decreased intake of vegetables compared to their food-secure peers because of reduced availability [28]. However, research by Poulsen and colleagues (2019) found that food-security status was not associated with vegetable consumption [12]. Further research is needed to determine the relationship between home vegetable availability and intake among food-insecure children. Our research further explains the nuance of the relationship between household food insecurity and vegetable availability. This study found a significant association between the availability of vegetables and food security, with food-insecure households reporting decreased availability.

Cost has been consistently found to be a major barrier to accessing and purchasing fresh vegetables for low-income individuals [21,29,30,31,32]. Qualitative research has reported that low-income households may avoid purchasing vegetables even when available because of the lack of high-quality options [31,33]. Efforts to increase access to fresh produce have resulted in a growing number of vegetable access programs. A recent systematic review reported that the introduction of new food retail opportunities within low-income communities that sell fruit and vegetables may provide a means of accessing fresher and better-quality produce and have more beneficial impacts on overall diet for low-income and food-insecure households compared to traditional grocery stores and supermarkets [34]. Community gardens [35,36,37] and farmers’ markets [38,39,40], especially those that accept food assistance benefits, have been shown to increase food security and vegetable intake. These programs are most likely to succeed when they simultaneously address multiple barriers to access or when coupled with other interventions or strategies [31].

This study found a null effect for SNAP participation and vegetable availability. There is some research that suggests that receipt of SNAP benefits in addition to associated vegetable incentive programs may increase purchase and consumption of vegetables [41,42]. However, other research has highlighted that recipients still experience incentive eligibility barriers or are unaware that these programs are available to them [31,43,44]. Local and federal policies can be utilized to remove or lower incentive eligibility, purchasing hurdles, and access barriers.

While it might be expected that barriers to buying vegetables drive a lower home availability of vegetables in low-income families, barriers to purchasing vegetables were not associated with the home availability of vegetables in our study. While availability was not impacted by access barriers, food-insecure households still reported experiencing access barriers to a greater degree than food-secure households. Emerging research has found that the extent to which differential access to healthy foods is thought to explain nutritional inequalities between low-income and high-income households may be overstated. Differences in nutrition knowledge, preferences, and tastes may have a greater impact than the retail environment [45]. The observed null effect may also be due to the attenuation of the preparing/cooking vegetables barriers score within our model.

This study found that barriers to preparing/cooking vegetables were more likely to be reported in food-insecure households compared to food-secure households. Additionally, this study also found that households that experienced increased barriers to preparing/cooking vegetables were more likely to be associated with a lower home availability of vegetables. These barriers encompass aspects tied to practical food knowledge and skills [46]. A greater amount of time spent on home food preparation is associated with an increased vegetable intake [47]. In the United States, the majority of meals are consumed away from the home [48]; however, there is some research to suggest that cooking at home has increased in recent years [49]. Research has found that food-insecure individuals have a similar frequency of cooking compared to their food-secure peers [50]. However, meals in food-insecure homes are less complex, which may be due to less time spent planning meals or having limited food preparation equipment [27,51].

Lack of meal planning and cooking complexity may also be a result of decreased knowledge and skills in relation to food and nutrition. A study by Begley and colleagues (2019) reported that limited food literacy was associated with greater food insecurity [23]. Knowledge and skills in relation to food and nutrition are a targetable outcome for nutrition education, as they can improve through education and skill development [52]. Prior research has shown that children involved in cooking activities have a higher vegetable intake compared to children who do not help [53,54,55]. Educating parents on successful ways to utilize their child’s help, coupled with equipping children with basic cooking skills, may be a potential strategy to overcome this barrier.

This study has several limitations. First, because of the cross-sectional design, only associations, not causal relationships, can be inferred. This study focused on the barriers from store to household that are present for households. These challenges are most likely not unique to our study’s population. However, a firm understanding of unique or different population-specific determinants and barriers to intake that may exist is required when developing tailored interventions. While this study controlled for a number of demographic, psychosocial, behavioral, and socio-environmental factors, additional factors may influence this relationship and be critical in guiding future public health efforts.

To adequately address food insecurity and improve vegetable consumption, multilevel strategies that provide additional resources at the individual, household, community, and system levels are needed [22,56]. Future interventions should consider all four of the pillars of food security (availability, access, utilization, and stability) [15,16]. This study in particular highlights the need for household-level interventions that focus on overcoming the barriers to preparing and cooking vegetables. These interventions should be delivered in conjunction with food assistance programs or food relief strategies that provide greater financial resources for food within the household [22]. Public health professionals possess the education and competency to make valuable contributions to overcoming barriers and improving the availability of vegetables within food-insecure populations.

## Figures and Tables

**Table 1:** Questionnaire items included in the TX Sprouts parent questionnaire assessing availability and barriers to buying and preparing/cooking vegetables.^1^

	Questionnaire Items	Response Options
Availability of vegetables at home	What foods were available in your home last week? Fresh vegetables Canned, frozen, or dried vegetables Salad Cut-up fresh vegetables in a place that is easy for kids to reach	All of the time Most of the time Some of the time Never
Barriers to buying vegetables	Do you experience any of the following challenges when buying vegetables for meals in your home? Vegetables are too expensive I cannot find quality vegetables The stores near me do not sell fresh vegetables	Yes No
Barriers to preparing/cooking vegetables	Do you experience any of the following challenges when preparing or cooking vegetables in your home? It is hard to use fresh vegetables before they spoil My family does not like vegetables I do not have time to prepare vegetables I do not know how to prepare vegetables I do not have simple and quick recipes My family does not help me cook	Yes No

^1^ Questionnaire items were adapted from those used by Evans A., et al. [26].

**Table 2:** Distribution of reported barriers to buying and preparing/cooking vegetables between food-secure and food-insecure households.

Questionnaire Items and Responses	Food-Secure (*n* = 1443)	Food-Insecure (*n* = 538)	*p*-Value ^a^
**Barriers to Buying Vegetables for Meals**			
Vegetables are too expensive			<0.001
Yes	135	232	
No	1308	306	
I cannot find quality fruits and vegetables			<0.001
Yes	93	70	
No	1350	468	
The stores near me do not sell fresh fruits and vegetables			0.108
Yes	25	16	
No	1418	522	
**Barriers to Preparing or Cooking Vegetables**			
It is hard to use fresh vegetables before they spoil			<0.001
Yes	192	115	
No	1251	423	
My family does not like vegetables			0.022
Yes	178	88	
No	1265	450	
I do not have time to prepare vegetables			<0.001
Yes	36	34	
No	1407	504	
I do not know how to prepare vegetables			<0.001
Yes	81	57	
No	1362	481	
I do not have simple quick recipes			<0.001
Yes	187	140	
No	1256	398	
My family does not help me cook			<0.001
Yes	47	47	
No	1396	491	

^a^*p*-value from χ^2^.

**Table 3:** Mixed-effects linear regression model of the impact of barriers to buying and preparing/cooking vegetables on the home availability of vegetables score in food-secure and food-insecure households.

Variable	Unstandardized β	Standard Error	95% CIs for β	*p*-Value
**Race/Ethnicity**				0.001
Non-Hispanic white	Referent	—	—	—
Hispanic	−0.56	0.23	−1.00, −0.12	0.01
Non-Hispanic African American	−0.35	0.15	−0.64, −0.06	0.02
Other	−1.14	0.32	−1.76, −0.51	<0.001
**SNAP Benefits**				0.645
No	Referent	—	—	—
Yes	0.06	0.13	−0.19, 0.31	0.645
**Food-Security Status**				<0.001
Food-secure	Referent	—	—	—
Food-insecure	−1.18	0.14	−1.45, −0.92	<0.001
**Parent Education**				0.03
Less than a high school diploma	Referent	—	—	—
High school diploma	−0.08	0.16	−0.39, 0.24	0.63
Some college or greater	0.30	0.15	0.01, 0.60	0.05
**Number of Children in the Home**	−0.05	0.05	−0.16, 0.05	0.30
**BB Score**	−0.19	0.11	−0.41, 0.03	0.09
**PCB Score**	−0.77	0.06	−0.88, −0.65	<0.001

Abbreviations: CIs, confidence intervals; SNAP, Supplemental Nutrition Assistant Program; BB, buying barriers; PCB, preparing/cooking barriers.
